# Transient Growth Arrest in *Escherichia coli* Induced by Chromosome Condensation

**DOI:** 10.1371/journal.pone.0084027

**Published:** 2013-12-23

**Authors:** Andrea L. Edwards, Dipen P. Sangurdekar, Kyeong S. Jeong, Arkady B. Khodursky, Valentin V. Rybenkov

**Affiliations:** 1 Department of Chemistry and Biochemistry, University of Oklahoma, Norman, Oklahoma, United States of America; 2 Department of Biochemistry, Molecular Biology and Biophysics, University of Minnesota, St Paul, Minnesota, United States of America; Universität Stuttgart, Germany

## Abstract

MukB is a bacterial SMC (structural maintenance of chromosome) protein that regulates the global folding of the *Escherichia coli* chromosome by bringing distant DNA segments together. We report that moderate overproduction of MukB may lead, depending on strain and growth conditions, to transient growth arrest. In DH5α cells, overproduction of MukB or MukBEF using pBAD expression system triggered growth arrest 2.5 h after induction. The exit from growth arrest was accompanied by the loss of the overproducing plasmid and a decline in the abundance of MukBEF. The arrested cells showed a compound gene expression profile which can be characterized by the following features: (i) a broad and deep downregulation of ribosomal proteins (up to 80-fold); (ii) downregulation of groups of genes encoding enzymes involved in nucleotide metabolism, respiration, and central metabolism; (iii) upregulation of some of the genes responsive to general stress; and (iv) degradation of the patterns of spatial correlations in the transcriptional activity of the chromosome. The transcriptional state of the MukB induced arrest is most similar to stationary cells and cells recovered from stationary phase into a nutrient deprived medium, to amino acid starved cells and to the cells shifting from glucose to acetate. The *mukB*++ state is dissimilar from all examined transcriptional states generated by protein overexpression with the possible exception of RpoE and RpoH overexpression. Thus, the transcription profile of MukB-arrested cells can be described as a combination of responses typical for other growth-arrested cells and those for overproducers of DNA binding proteins with a particularly deep down-regulation of ribosomal genes.

## Introduction

MukBEF protein complex is a bacterial prototype of eukaryotic condensins and cohesins and plays a central role in global folding of the *Escherichia coli* chromosome [1], [2], [3], [4], [5]. Purified MukBEF promotes ATP-stimulated chirality-sensitive DNA condensation in a mechanism that can produce a network of multi-molecular protein clamps on DNA [3], [6], [7], [8], [9]. All DNA reshaping activities of the complex reside in its core SMC (structural maintenance of chromosomes) subunit MukB, whereas MukEF regulates MukB-DNA interactions [3], [7], [10], [11] and recruits MukBEF into clusters at the center of the nucleoids [11], [12], [13], [14].

The three subunits of MukBEF are encoded within operon *smtA-mukF-mukE-mukB* together with an unrelated gene *smtA* [2]. Mutational inactivation of MukBEF results in chromosome decondensation and cutting, anucleate cell formation and a severe decline in colony formation above 30°C [2], [15], [16]. Conversely, overproduction of MukB or MukBEF leads to chromosome condensation [16]. The overproduced MukB can rescue the chromosome decondensation phenotype of MukEF mutants but not their temperature sensitivity or anucleate cell formation [2], [16].

The copy number of MukB increases from about 400 per exponentially growing cell to about 1,000 during stationary phase [3], suggesting that MukBEF might contribute to the onset of stationary phase in bacteria. For comparison, eukaryotic condensins have been implicated in establishment of chromosome-wide gene silencing presumably by inducing chromosome condensation [17]. However, mild constitutive overproduction of MukBEF has no discernible effect on the cell physiology. In contrast, a 100-fold overproduction of MukB of MukBEF induces chromosome condensation, which is followed by growth arrest [16]. Here, we explored this growth arrest in greater details.

We found that the growth arrest induced by overproduction of MukB or MukBEF (but not of SmtA or MukEF) is transient and occurs in a strain- and growth conditions-dependent manner. In further distinction from other overexpression experiments [18], [19], [20], this growth arrest was delayed and only modestly affected cell viability. The cells adapted to the ensuing excessive chromosome condensation and eventually resumed their growth. The exit from growth arrest was accompanied by the loss of excessive chromosome condensation and the overproducing plasmid.

DNA microarray analysis of arrested cells revealed marked downregulation of ribosomal and biosynthetic genes and upregulation of a variety of stress response proteins. Compared to the control culture, more than 400 genes were affected by the overproduction of the condensin. The resultant profile shared significant similarity with those for non-growing and starving cells. However, the extent of repression of the chromosomal locus encoding ribosomal proteins was one of the highest among all publicly available datasets. Accordingly, the genes with altered expression were markedly enriched in this locus but randomly distributed throughout the rest of the chromosome.

## Materials and Methods

### Plasmids and growth media

MukB, MukBEF, MukEF, and SmtA were produced from plasmids pBB10, pBB03, pBB08, and pBB05, which contain, respectively, *mukB-His10*, complete *smtA-mukF-mukE-mukB-His10* operon, *smtA-mukF-mukE-His9*, or *smtA* under the control of arabinose-inducible promoter P_BAD_ [6], [11]. Protein expression was induced by the addition of 0.2% arabinose, which results in saturating protein production in the entire population of cells. As a control, we used the vector alone, pBAD-myc/HisB (Invitrogen). The following growth media were used: LB containing 0.5% NaCl; M9 medium containing 2 mM MgSO_4_, 0.1 mM CaCl_2_, 100 µg/ml thiamine, 0.2% glycerol; and M9CA, which is M9 medium supplemented with 0.4% Casamino Acids (Difco).

### Cell growth and fluorescence microscopy

Overnight cell cultures were grown in LB at 37°C, washed in prewarmed medium and inoculated into fresh growth medium. At OD_600_ of 0.2, 0.2% arabinose was added to induce the overproduction of proteins. At appropriate time points, cell aliquots were fixed with 70% ethanol, stained with 100 nM DAPI, 1xSyproOrange (Molecular Probes) and observed using Olympus BX-50 microscope [16]. The size of cells and nucleoids was quantified using software Nucleus [16].

### Microarray analysis

Cells with the *mukB* gene under the control of arabinose-inducible promoter were collected at 0, 2.5, 5 and 7.5 h after addition of arabinose or glucose. Transcript levels in sugar treated cultures were assessed relative to those before addition (0 h; OD_600_ equal to 0.2) of the inducer or repressor. The relative transcript abundances were determined for more than 98% of all annotated *E. coli* ORFs using whole-genome DNA microarray as described previously [21], [22]. Following removal of systematic biases in microarray experiments [23], at least three replicate measurements of relative mRNA abundances were used to infer differential expression between arabinose and glucose treated samples. The inferences were done using a *t*-test corrected for multiplicity of measurements at a false discovery rate (FDR) of less than 5%. Estimated relative transcript abundances for individual genes were pooled within annotated gene classes (http://genprotec.mbl.edu/) and univariate statistics were derived for respective functional categories of genes.

Gene set analysis was done by (i) computing a weighted expression metric for each individual gene set, (ii) normalizing the metric under two null hypothesis as described by Tian et al [24] and (iii) computing the p-value and q-value for the normalized gene set score [25]. For computing the normalized score, a compendium of *E. coli* expression data (OU GeneExpDB) comprising of over 1,000 arrays was used. The resulting normalized set scores can be compared within the same condition and also within conditions [25]. Local False Discovery Rates (LFDR) and posterior probability scores [26] was computed for the set scores, and posterior probabilities greater than 90% (corresponding to FDR of less than 5%) were considered significant.

## Results

### Overproduction of MukBEF and MukB triggers a transient growth arrest

**Figure 1:**
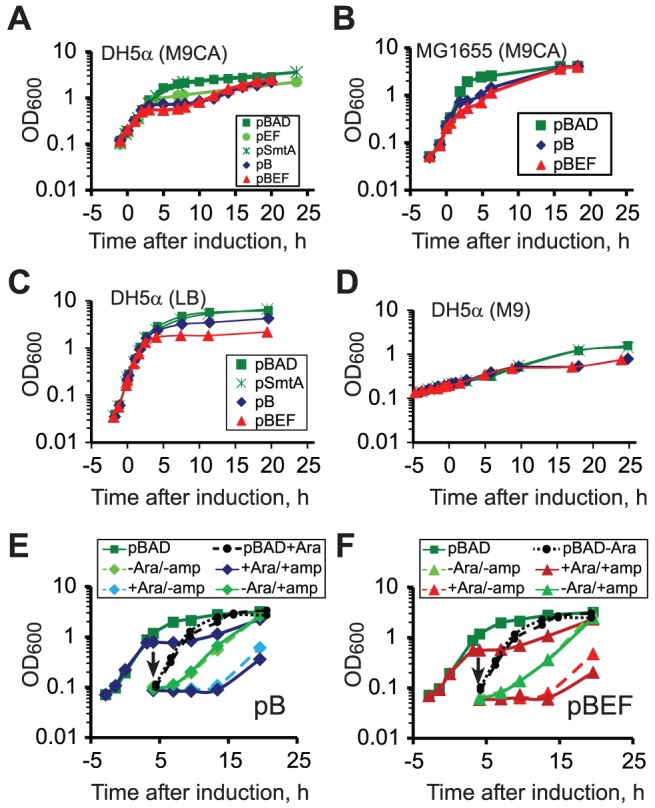
Overproduction of MukBEF and MukB induces transient growth arrest. Shown are the average growth curves (*n* between 2 and 8; SEM are shown when bigger than symbols) obtained after induction of MukB (pB), MukBEF (pBEF), MukEF (pEF) or SmtA (pSmtA). The cells harboring vector alone (pBAD) were used as a control. (A) DH5α cells grown in M9CA. (B) MG1655 cells grown in M9CA. (C and D) DH5α cells grown in LB and M9. (E and F) Growth arrest is not caused by a secreted factor. Aliquots of arrested DH5α cells harboring pBB10 (C) or pBB03 (D) were washed and inoculated into the fresh medium, which was either supplemented or not with 0.2% arabinose (+Ara or –Ara) or 50 µg/ml ampicillin (+amp or -amp).

We first explored effects of MukBEF overproduction on *E. coli* growth under various conditions. DH5α cells harboring appropriate plasmids were grown in M9CA medium at 37°C up to OD_600_ of 0.2, protein overproduction was induced by the addition of 0.2% arabinose and the turbidity of cell culture was then followed (Figure 1). Under these conditions, overproduction of MukBEF or MukB results in abrupt growth arrest 2.5 hours after induction, presumably due to a direct action of the proteins on DNA [16]. Accordingly, no plateau in the growth curves was detected upon overproduction of SmtA and MukEF, which do not bind DNA, although some slowdown in cell growth could be observed upon overproduction of MukEF (Figure 1A). Curiously, we found that both MukBEF and MukB overproducers resumed growth about nine hours after induction with a lower growth rate (Figure 1A). The resumption of cell growth was not the result of microbial contamination since the growth curves were highly reproducible over several independent experiments and were not affected by complete removal of ampicillin from the growth medium (Figure 1 and data not shown).

The phenotype of growth arrest was medium and strain dependent. We observed a clear growth arrest for DH5α cells that overproduced MukBEF or MukB but not SmtA in M9 medium (Figure 1D). In LB, the arrest occurred at higher turbidity, about one cell division prior to the entrance into the stationary phase, and no exit from it could be detected for at least several hours (Figure 1C). Overproduction of MukBEF and MukB in MG1655 cells produced an abrupt decline in the growth rate, although did not completely block cell division (Figure 1B). We conclude therefore that overproduction of condensins elicits a distinct change in physiological state of the cells.

### Growth arrest is not maintained by a secreted factor

The arrested cells did not immediately resume growth after we diluted them into the fresh medium. In this experiment, 3 ml of arrested cells was pelleted, washed and resuspended in prewarmed M9CA medium, and then inoculated into 20 ml of M9CA medium, which was either supplemented or not with 0.2% arabinose and 50 µg/ml ampicillin (Figure 1E, F). If arabinose was omitted from the fresh M9CA medium, the cells resumed growth 7 h after induction, about the same time as the undiluted cells. In the presence of arabinose, the resumption of cell growth occurred later, 13 h after induction. Afterwards, the cells were growing somewhat faster if ampicillin, the selection marker for the plasmids, was omitted from the growth medium indicating that the plasmids could be lost faster at high concentrations of condensin.

In all cases, the cells did not resume growth for at least three hours after dilution. In contrast, the non-overproducing cells resumed their growth immediately after dilution (Figure 1E, F). We conclude that the arrested state is not maintained by a secreted factor (which would be removed once the growth medium was replaced) and is not sensitive to the optical density of the culture. Rather, the arrested state is an intrinsic physiological state of bacteria.

### Colony forming ability of arrested cells is moderately reduced

**Figure 2:**
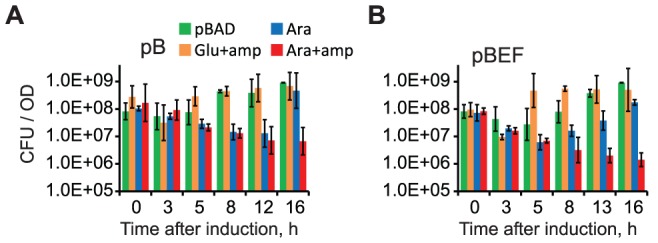
Reduced viability of arrested cells. DH5α cells harboring the vector (pBAD), pBB10 (A) or pBB03 (B) were grown in M9CA medium up to OD_600_ of 0.2, supplemented with 0.2% glucose (Glu) or 0.2% arabinose (Ara) and tested for colony formation on LB plates containing 0.2% glucose with or without 100 µg/ml ampicillin, as indicated. DH5α(pBB10) and DH5α(pBB03) cells grown with glucose showed no difference in colony formation when plated with or without the drug (data not shown). The average (± S.D.) of two to six separate measurements of colony forming units (CFU) is shown.

Colony forming ability of arrested cells was measured by plating on LB agar supplemented with 0.2% glucose in the presence or absence of 100 µg/ml ampicillin. As a control, we used DH5α(pBB10) and DH5α(pBB03) cells grown in the presence of 0.2% glucose and DH5α(pBAD) cells grown in the presence of 0.2% arabinose. As expected, the cells in all control cultures had the same colony forming ability regardless of whether they were plated with or without ampicillin (Figure 2 and not shown). The colony formation of cells that overproduced MukB or MukBEF declined by about 50-fold compared to the control cultures and was the lowest during the growth arrest. The colony formation recovered once cells exited the arrest phase. In this case, however, the majority of colonies did not contain the ampicillin resistance marker (Figure 2). Thus, the exit from growth arrest is accompanied by the loss of the plasmid. Notably, the plasmid loss occurred after cells resumed their growth, indicating that it was due to impaired plasmid partitioning or replication.

### Growth arrest correlates with chromosome condensation

**Figure 3:**
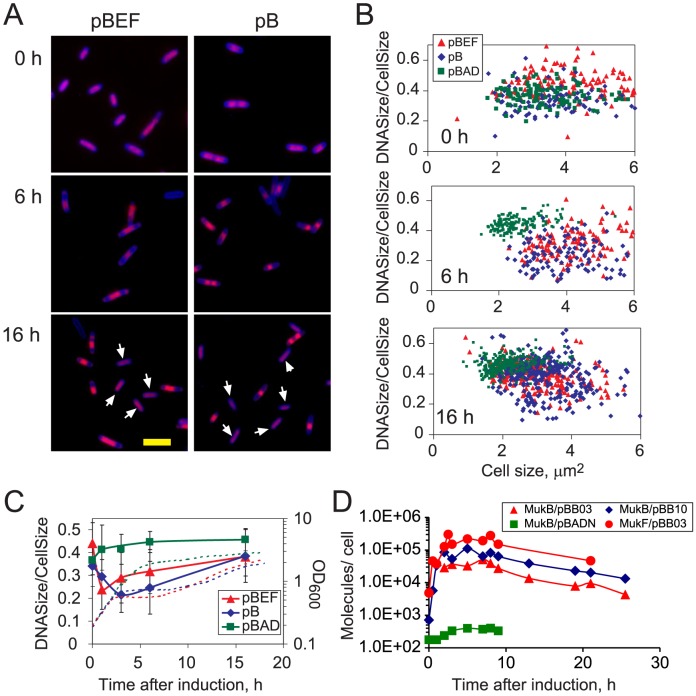
Growth arrest parallels chromosome condensation. DH5α cells harboring either the vector (pBAD), or pBB10 (pB), or pBB03 (pBEF) were grown in M9CA medium, supplemented with 0.2% arabinose at OD_600_ of 0.2 and analyzed by fluorescence microscopy. (A) Overlays of DAPI fluorescence (red) and SyproOrange fluorescence (blue) for cells at 0, 6 and 16 hours after induction. The arrows point out cells with uncondensed chromosomes. Scale bar, 5 µm. (B) The extent of chromosome condensation plotted as a function of cell size. (C) The extent of chromosome condensation at different times after the addition of arabinose. For each time point, the average (± S.D.) for at least 100 cells is shown. The dashed lines show the growth curves from Figure? 1A. (D) The copy number of MukB and MukF in cells that overproduce MukB or MukBEF (assuming 10^9^ cells per OD) measured using quantitative immunoblotting as previously described [16].

We next examined the extent of chromosome condensation in the arrested cells using fluorescence microscopy. As reported earlier [16], the overproduction of MukBEF and MukB induced chromosome condensation in DH5α cells as early as 1 h after induction, before the growth arrest (Figure 3). The chromosomes remained condensed throughout the growth arrest. However, following resumption of growth, two populations of cells could be found. In one, chromosomes were condensed. In the other population, chromosomes were of normal size (Figure 3A).

We next confirmed the existence of two populations by quantifying the size of the chromosomes in cells from various growth phases. In Figure 3B, the extent of chromosome condensation is plotted against the size of the corresponding cells. Prior to protein induction, the distributions of cells that carried pBB03, pBB10 or pBAD plasmids are the same. By 6 h after induction, the control culture, which approached the stationary phase, consisted mostly of short cells. The arrested cells remained long and contained smaller chromosomes. By 16 h after induction, many cells that overproduced MukB or MukBEF were short and contained normal size chromosomes. This result was confirmed by quantifying the average chromosome size throughout the entire population of cells (Figure 3C). Chromosome condensation reached its peak by the onset of growth arrest and gradually dissipated thereafter.

The loss of excessive condensation suggested that the cells that exit the growth arrest state limit somehow overproduction of condensins. Using quantitative immunoblotting, we indeed found that the amount of MukB remains constant during the growth arrest both in MukB and MukBEF overproducers but steadily declines thereafter (Figure 3D). Similar pattern was observed for the amount of MukF in cells that overproduced MukBEF. This decline in protein content parallels the loss of ampicillin resistance (Figure 2). This also supports our conclusion that plasmid loss accelerates during growth arrest.

### Transcriptional signature of growth arrest

To identify condensin-specific features of the arrest, we next analyzed transcriptional states of the bacteria as detailed in Material and Methods. The response at each time point (2.5 h, 5 h, and 7.5 h of exposure to arabinose inducer) was characterized by determining all significantly affected gene sets. Sets that were more than 90% likely to be differentially expressed were considered significant. At this cut-off, 16 unique gene sets were affected at the first time point, 61 – at the second, and 21 – at the third (Table S1). Although most of the sets were down-regulated, over-production of MukB, whose transcript was induced 47±8 - fold compared to 1.5±0.6 - fold in the no-arabinose control, did result in significant induction of the sets of the stress response genes. Also significantly upregulated by the inducer were, expectedly, the arabinose catabolism genes.

### Patterns of gene set activity in *mukB*++ condition

**Figure 4:**
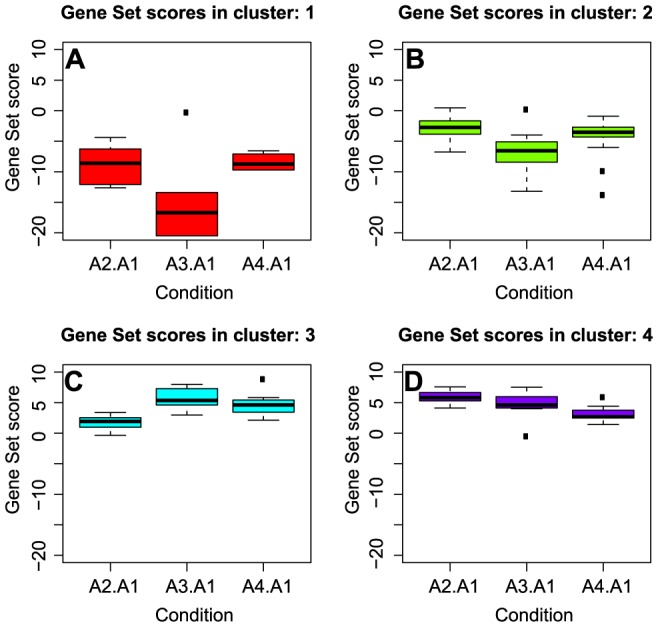
Patterns of gene set activity during MukB induction. Gene set scores computed using gene expression responses after 2.5(A2), 5 h (A3) and 7.5 h (A4) post MukB induction, were sorted based on similarity of profiles into four clusters. Clusters 1 and 2 (panels A and B) are characterized by lower gene set scores as compared to pre-treatment control, with maximum perturbation during the 5 h time point. Clusters 3 and 4 (panel C and D) represents gene sets which are induced on average during MukB induction. Cluster 3 gene sets show a positive trend with time of induction, whereas cluster 4 sets exhibit a steady decline in set scores with time.

All gene sets which were significantly affected in at least 1 out of 3 points during the arrest (Table 1) were classified into four clusters based on magnitude and direction of the response (Figure 4). Most repressed sets were those associated with protein and nucleotide synthesis (Panel A). Sets composed of pathways related to respiration, energy production, central carbon metabolism and supercoiling (Panel B) were also repressed, but to a lesser extent. Targets of transcription factors RpoH, RpoN, ExuR and PspF and membrane components were mildly upregulated, peaking at 5 h post induction (Panel C). At the same time, arabinose transport and metabolism were induced in arrested *E. coli*, along with targets of CRP (Panel D). Thus MukB-induced arrest can be primarily characterized by a severe downregulation of gene classes whose products are either involved in energy production or heavily dependent on a supply of energy equivalents, most notably protein biosynthesis Of note, downregulation of genes encoding components of ribosomes 5 h after induction of MukB is the second deepest of all conditions examined.

**Table 1:** List of gene sets active in at least one of the 3 *mukB*++ conditions.

1.1.1_Carbohydrates/Carbon_compounds_
1.1.1.8_L-arabinose_catabolism_
1.1.3.5_Glycine_cleavage_
1.3.1_Glycolysis_
1.3.2_Pentose_phosphate_shunt,_oxidative_branch_
1.3.3_Pyruvate_dehydrogenase_
1.3.4_Tricarboxylic_acid_cycle_
1.3.6_Aerobic_respiration_
1.3.7_Anaerobic_respiration_
1.3.8_ATP_proton_motive_force_interconversion_
1.4.1_Electron_donor_
1.5.1.10_Glycine_
1.5.1.16_Histidine_
1.5.2.1_Purine_biosynthesis_
1.5.2.2_Pyrimidine_biosynthesis_
1.7.3_Pentose_phosphate_shunt,_non-oxidative_branch_
1.7.8_Gluconeogenesis_
1.7.17_Formyl-tetrahydrofolate_biosynthesis_
1.7.21_Glyoxylate_degradation_
1.7.35_Lactate_oxidation_
2.2.2_Transcription_related_
2.2.5_tRNA_
2.2.6_rRNA,_stable_RNA_
2.3.1_Amino_acid-activation_
2.3.2_Translation_
2.3.8_Ribosomal_proteins_
3.1.2.3_Repressor_
3.1.3.1_Translation_attenuation_and_efficiency_
4.1.B.1_The_General_Bacterial_Porin_(GBP)_Family_
4.3.A.1.m_membrane_component_
4.3.A.2_The_H+/Na+-translocating_F-,_V-_and_A-type_ATPase_(F-ATPase)_Superfamily_
4.3.D.1_The_Proton-_or_sodium_ion-translocating_NADH_Dehydrogenase_(NDH)_Family_
4.3.D.4_The_Proton-translocating_Cytochrome_Oxidase_(COX)_Superfamily_
4.S.82_H+_
4.S.100_L-arabinose_
4.S.119_methylgalactoside/galactose_
4.S.130_Na+_
5.5.2_Temperature_extremes_
6.6_Ribosome_
8.1_Prophage_genes_and_phage_related_functions_
10_cryptic_genes_
Supercoiling_sensitive_genes
AraC
ArcA
ArgP
CRP
DnaA
EnvY
ExuR
FNR
Fis
FruR
Fur
GadE
GalR
GalS
GcvA
KdpE
Lrp
MarA
PdhR
PspF
PurR
RpoE
RpoH
RpoN

### Comparison with compendium of gene expression profiles

To determine whether and to what extent such transcriptional response is unique to this condition, we compared the signature responses of *mukB*++ cells with signature responses in the compendia of publicly available data sets (via OU GeneExpDB http://genexpdb.ou.edu/main/). To evaluate similarities between conditional responses, we computed Spearman's Rank correlation between the set scores of *mukB*++ profiles and profiles of each individual condition within the data set. To assess the significance of the correlation, we permuted the set scores for each condition and computed the Spearman's rank correlation coefficient under the null hypothesis that the observed correlation coefficient is no different than if sampled randomly from a null distribution. Each of the *mukB*++ profiles was correlated with the other two, as expected, with the high median correlation value of 0.83. While each of the *mukB*++ conditions was also observed to be significantly correlated (FDR<1%) with about 350 of over 1050 conditions in our compendium, we next focused on the top 30 correlated conditions (R>0.45 for the three *mukB*++ profiles).

The *mukB*++ transcriptional state appears to be qualitatively similar to the states in three broad categories of conditions: starvation, nutrient or conditional shifts, and carbon diauxie. The similar starvation responses, relative to the transcriptomes of exponentially growing cells, include: (i) transitioning of deeply starved cells into buffered salt medium without any nutrients (ii) diauxic shift of cells from glucose to acetate as carbon source, (iii) shift from aerobic to anaerobic conditions in exponential growth phase, (iv) late stationary phase, more than 24 hours, in rich medium; and (v) a single amino acid starvation.

**Figure 5:**
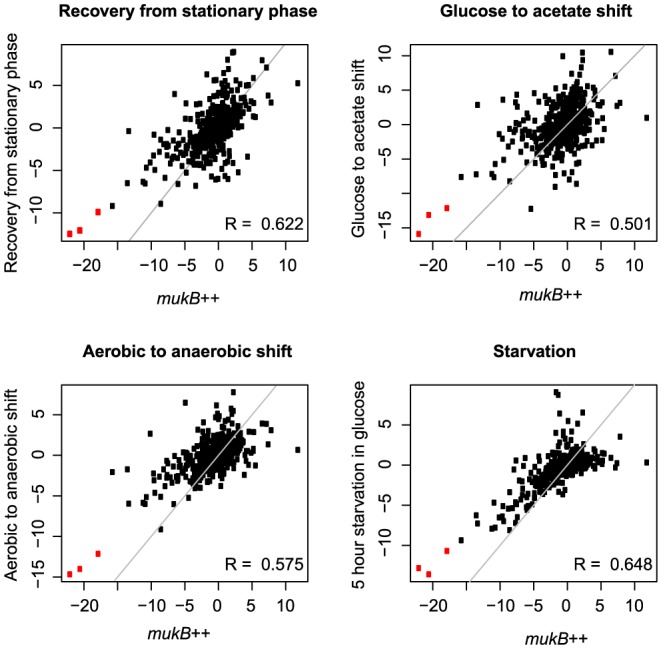
Correlation of gene set scores with representative similar conditions. Shown are scatter plot of gene set scores of conditions most similar to MukB induction (5 h time point) based on Rank correlation, which include 75 min recovery from stationary phase in sodium phosphate medium, 1 h after shift from glucose to acetate medium, 1 h shift from aerobic to anaerobic growth conditions and 5 h starvation in glucose medium. Set scores along the diagonal have the same values in these conditions. For comparison, the four reference conditions are correlated with each other with median Spearman correlation of 0.43. Sets related to ribosomal proteins and translational apparatus (indicated in red) are more downregulated in *mukB*++ condition than in the other conditions.

**Figure 6:**
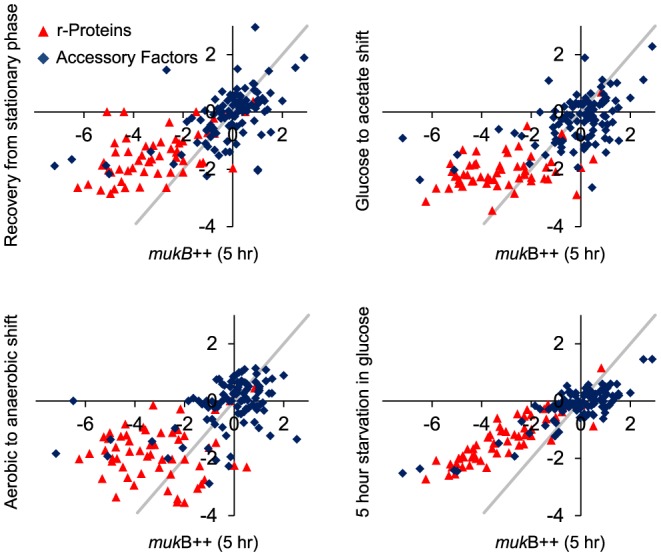
Expression of protein synthesis genes in representative similar conditions. Genes encoding ribosomal proteins are significantly more down-regulated in *mukB*++ samples than in representative similar conditions, as indicated by their lower log (base 2) expression values.

To visualize similarities between *mukB*++ and representative conditions, we generated scatter plots of corresponding pairs of gene set scores of these conditions and the 5 h sample of MukB induction (Figure 5). Despite overall correlation and score magnitude consistency, several outlier sets were readily identifiable. In all the conditions– recovery from stationary phase into a phosphate buffer, diauxic shift from glucose to acetate as carbon source, shift from aerobic to anaerobic growth conditions and prolonged starvation in glucose media– the ribosomal proteins and translational apparatus are relatively less downregulated than in *mukB*++ condition. Detailed analysis of gene expression in the “protein synthesis- protein modification” pathway revealed up to 16-fold deeper downregulation of ribosomal proteins in *mukB++* cells compared to the reference conditions (Figure 6). Other excessively downregulated proteins included the major translation factors TufA, TufB, FusA, Tsf and LepA. In diauxic shift from glucose to acetate, iron responsive transcriptional regulator Fur is highly downregulated compared to the *mukB*++ condition. In all four cases, the extracytoplasmic stress responsive PspF regulator set is highly expressed in *mukB*++ compared to the other conditions. In prolonged starvation in glucose medium, tryptophan synthesis is upregulated compared to the *mukB*++ condition.

The comparison across conditions also revealed that among almost 40 available protein over-expression profiles, only profiles generated by over-expression of RpoE and RpoH [27], [28] were significantly similar to *mukB*++ (R>0.50). This is noteworthy in light of the fact that many of the reported over-expression experiments resulted in the cessation of growth, similar to *mukB*++.

Thus, transcriptional response in *mukB++* cells was dominated by cessation of metabolic activity as it develops during deep starvation or overproduction of DNA binding proteins, with very few features that could be uniquely attributed to condensins.

### Suppression of spatial correlations in gene expression after growth arrest

**Figure 7:**
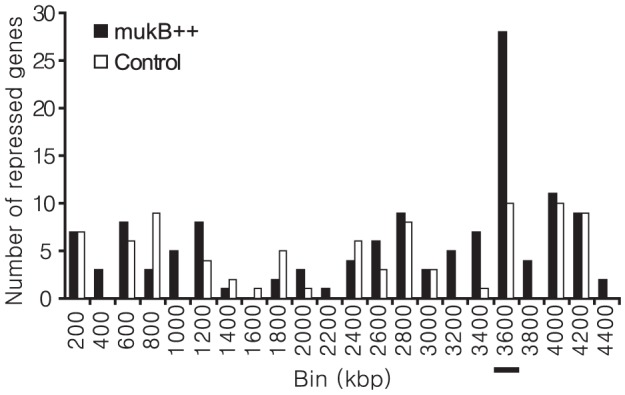
Distribution of the differentially repressed genes in *mukB++* cells at 2.5 h after induction. The 138 and 90 genes were identified as significantly differentially repressed more than 2-fold in the *mukB++* and control cells, respectively. Since at one extreme all 138 repressed genes could be co-localized and at another extreme they can be uniformly distributed among 30 (4200/138) evenly spaced bins on the chromosome, the bin size of 200 consecutive ORFs was chosen as closest, round upper bound that accommodates both possibilities. The repressed genes in *mukB*++ cells are most frequently found in the bin from 3400 to 3600 kb. The bin contains the largest spatial cluster of ribosomal genes, 29 ribosomal genes are situated between 3408 to 3472 kb (underlined).

With a notable exception of ribosomal genes, the genes affected by MukB overproduction appeared to be randomly distributed along the *E. coli* chromosome (Figure 7). Such distribution is consistent with the view of non-specific binding of the protein to DNA, which randomly interferes with gene expression. This conclusion was further supported by the analysis of spatial correlations in gene expression in the overproducing cells

**Figure 8:**
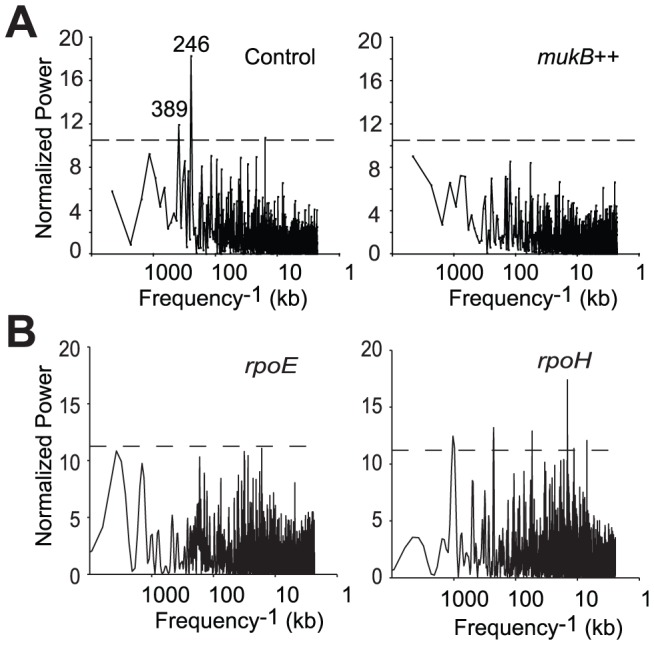
The Lomb periodogram of transcriptional activity. (A) Spatial series of changes in relative transcript abundances at 2.5 h after induction relative to the time zero (right) or repression (left) of MukB were analyzed as described elsewhere [29]. The frequencies that cross the 95% confidence level (horizontal line) were defined as significant. (B) Frequency analysis of transcriptional changes during overproduction of RpoE [27] and RpoH [28] at the longest tested time points, 60 min and 20 min after induction, respectively.

Spatial analysis of global transcription can be used to relate the state of the chromosome, such as the DNA supercoiling, to the state of transcriptional activity [29]. This approach stems from the realization that the folding of the chromosome into a higher-order structure may create regions similarly accessible to RNA polymerase on a scale that extends far beyond individual genes or operons. As a result, transcriptional activity of seemingly unrelated genes becomes correlated both at short and long intergenic separations [29], [30]. We indeed observed that transcriptional changes in the control, glucose culture proceeded in spatially coordinated manner during the onset of the stationary phase (Figure 8A). These changes could be characterized by pronounced long-range transcriptional patterns, including significant correlations at the 246^−1^ kb^−1^ and 389^−1^ kb^−1^ frequencies. In contrast, we were unable to detect any significant spatial transcriptional patterns in the *mukB++* cells. Instead, spatial gene expression correlations in the arrested cells were notably suppressed throughout the entire spectrum (Figure 8A).

The loss of the spatial co-regulation in transcription is not a general consequence of protein overexpression in *E. coli* since overproduction of other proteins, including RpoH and RpoE, did not diminish the statistically significant spatial patterns of transcriptional activity (Figure 8B). Rather, these data reveal massive changes in the structure of the chromosome of the arrested cells that abolished coordination in gene expression beyond transcriptional coregulation.

## Discussion

Protein overproduction in bacteria, especially using high yield expression systems, often arrests cellular growth. The case of MukB caught our attention because it occurred at relatively low levels of the protein (which was achieved by the rather mild saturating induction using pBAD system) and was transient in nature. Indeed, the copy number of the overproduced MukB was about 100,000 per cell (Figure 3D). This is on par with the endogenous levels of the nucleoid associated proteins FIS or HU, which are produced at 60,000 copies each [31], [32], [33]. For comparison, 40-fold overproduction of H-NS was reported to cause a transient growth arrest whereas similar overproduction of HU resulted in a 1.8-fold reduction in the growth rate but no cessation of growth [19]. This difference highlights the distinctive role of MukBEF in chromosome organization.

Another distinctive feature of this system is the long, 2.5-hour delay between induction of the protein and the onset of the growth arrest. In contrast, a variety of stresses- including cold shock, protein overproduction, peroxide treatment and nutrient depletion- result in immediate cessation of growth [34]. This delay was unlikely due to the buildup of the induced protein, which reached its plateau level within the first hour of induction (Figure 3D). Rather, it suggests that accumulation of MukB-dependent stress was needed to trigger growth arrest.

Notably, at the employed levels of protein induction, the arrest occurred only in DH5α cells whereas MG1655 cells slowed down their growth (Figure 1). Thus, severity of the effect depends on genetic make-up of the cell. Two mutations present in DH5α cells, *relA1* and *recA1*, deserve special attention due to their involvement in DNA metabolism. The *relA1* mutation is physiologically identical to *relA* null, both failing to produce ppGpp under conditions of amino acid starvation [35]. The *recA1* allele [36] used in the present study is deficient in all known functions of RecA, including ATPase activity, DNA binding, ATP-dependent conformational changes and repressor cleavage activity [37]. As a result, these *recA1* cells cannot activate RecA-LexA mediated SOS response and would not filament when exposed to DNA damage.

It seems unlikely, however, that these two mutations were directly responsible for the arrest. Indeed, since the alarmone ppGpp is required for starvation-dependent repression of translational machinery [38], the repression observed in *relA1 muk++* strains is not confounded (as it would in *relA* ^+^ *muk++*) by the stringent response and it strongly suggests an independent mechanism of global inhibition of transcription.

Similarly, *recA* mutation was shown to have no effect on expression of any gene in the *E. coli* genome, unless the cells are subjected to DNA damage [30], [39]. However, overproduction of MukB does not induce significant DNA damage as is evidenced by the lack of SOS-induced filamentation of the *recA1* DH5αand *recA^+^* MG1655 cells (Figure 3A and [16]) and similar levels of toxicity of MukBEF overproduction for *recA1* and *recA^+^* cells (Figure 2 and [16]). Higher levels of *recA*-mediated killing would be expected in *recA^+^* cells should they incur significant DNA damage. These data strongly argue that the mechanism of growth arrest occurs is largely independent of *relA* and *recA* functions.

Curiously, the viability of *mukB++* cells was only transiently affected, and, after a four-hour lag, the cells resumed their growth with a reduced rate. This growth pattern is similar to what is observed during the classic diauxic shift [40]. The adaptation to the excessive chromosome condensation involved a rapid loss of the overproducing plasmid. In this respect, the growth arrest served as a defense mechanism against an extrachromosomally produced DNA binding protein. A similar transient growth arrest has been previously observed upon overproduction of H-NS but not HU [19]. Perhaps the ability of H-NS and MukB to bridge DNAs underlies their ability to induce bacteriostasis.

In earlier studies, we observed that both transcript abundances and changes in transcript abundances are spatially correlated along the chromosome in at least one of three modes and/or their multiples: short-range (15–20 kbp), medium-range (110–130 kbp) and long-range (600–700 kbp) [29], [30], [41]. Conditions of normal growth and division produced all three types of correlations in spatial transcript abundances, whereas conditions of arrest or nutritional transitions usually resulted in complete or partial loss of short- and long-range correlations with the retention, and often amplification, of medium-range correlations.

Here, we observed that overexpression of MukB abolishes spatial correlation in the transcriptional signal to a greater extent than in any of the 41 examined protein over-expression sets (data not shown). This extensive degradation of spatial transcriptional patterns in *mukB*++ implies that the overexpression did not have a uniform effect on chromosome-wide transcription. Indeed, uniform down-regulation would have resulted in scaling down of the signal in the spatial domain but would not have affected significant frequencies following the Fourier transform. Non-uniformity of the effect was also evidenced by (i) the disproportionate down-regulation of genes involved in protein synthesis and and (ii) up-regulation of a number of stress response genes and genes involved in arabinose utilization on the backdrop of transcriptional inhibition of energy and growth related functionalities.

Physiological consequences of protein overexpression have been tackled in several investigations [20], [42], [43], [44], [45]. Those studies examined overexpression of heterologous soluble [44] and insoluble [44] proteins and artificial [42] polypeptides, endogeneous cytosolic [20] and membrane proteins [45]. Although specific questions and approaches varied across these studies, the majority of them revealed a slowdown or arrest of cellular growth, which was accompanied by the induction of RpoH controlled heat shock proteins that are involved in re-folding, degradation and aggregation of proteins. Overproduction of MukB triggered a similar response. Beyond that, *mukB*++ cells appear to be more similar to the slowing or arresting growth upon nutrient/energy down-shift or limitation. These data indicate that the similarity between diauxic shift and the growth arrest may go beyond the growth phenotype. Indeed, the fact that the transcriptional response of MukB over-production is significantly similar to transcriptional responses observed during glucose-to-lactate and glucose-to-acetate transitions suggests that the MukB induced arrest followed by recovery bears regulatory characteristics of the transient adaptive response.

One of the most dominant characteristics of the MukB overproduction response is an extensive and deep decline in the abundance of transcripts that encode ribosomal and translational proteins. Inhibition of translation has been reported to cause the lag during cold shock [34]. It is conceivable that the MukB-induced growth arrest employs a similar mechanism and that the arrest is ultimately triggered by silencing of the ribosomal genes that develops during induction of the condensin. In tentative support of this view, we found that the only chromosomal locus significantly enriched with the repressed genes encodes proteins involved in protein synthesis: 29 out of 64 genes encoding ribosome-associated proteins are localized between coordinates 3407092 and 3472574 on the chromosome and nearly all of them were significantly repressed when MukB was over-produced (Figure 7, “bin 3600”). The high affinity of MukB to single stranded DNA could ensure its preferred localization at the highly transcribed ribosomal genes, which are enriched in regions of melted DNA and rRNA. Such selective binding is not unlike the targeting of eukaryotic condensins to rDNA [46] and might reflect a common, highly conserved property of the proteins.

Of note, the state of MukB over-expression can be qualitatively differentiated from the states of RpoE and RpoH over-expression based on degree and consistency of repression of ribosomal protein genes and on the basis of spatial pattern formation. It is plausible that deep down-regulation of translational apparatus in *mukB*++ is incompatible with the formation of spatial correlations, or vice versa.

## Supporting Information

Table S1 **Genes whose expression was significantly affected during growth arrest.**
(XLSX)
